# Genome-Wide Identification and Analysis of Respiratory Burst Oxidase Homolog (*RBOH*) Gene Family in *Nelumbo nucifera*

**DOI:** 10.3390/ijms27188366

**Published:** 2026-09-19

**Authors:** Jinping Zou, Guo Ru, Yushi Zhang, Jie Zhang, Donghao Li, Kai Feng, Peng Wu, Shuping Zhao, Lei Shen, Liangjun Li

**Affiliations:** 1 School of Horticulture and Landscape Architecture, Yangzhou University, Wenhui East Road No.48, Yangzhou 225009, China; ping@yzu.edu.cn (J.Z.); rrruguo@163.com (G.R.); 15536609963@163.com (Y.Z.); zj199904@163.com (J.Z.); lidonghao0928@163.com (D.L.); 2 Joint International Research Laboratory of Agriculture and Agri-Product Safety of Ministry of Education of China, Yangzhou University, Yangzhou 225009, China; fengkai@yzu.edu.cn (K.F.); wupeng@yzu.edu.cn (P.W.); zhaoshuping@yzu.edu.cn (S.Z.); shenlei07@yzu.edu.cn (L.S.)

**Keywords:** *Nelumbo nucifera*, respiratory burst oxidase homolog, high-temperature stress

## Abstract

Respiratory burst oxidase homologs (RBOHs) are core enzymes for reactive oxygen species (ROS) production in plants and play critical roles in stress signal transduction. In this study, we performed a genome-wide identification of the *RBOH* gene family in *Nelumbo nucifera*, comprehensively employed phylogenetic analysis, gene structure analysis, cis-acting element prediction, and other bioinformatics approaches, and examined their expression patterns under high-temperature stress using qRT-PCR. A total of ten *NnRBOHs* were identified, unevenly distributed across eight chromosomes. Phylogenetic analysis classified them into five subfamilies, with members within the same subfamily sharing highly similar gene structures and conserved motifs. The promoter regions contained abundant cis-acting elements associated with hormone, light, and stress responses. Under heat treatment, *NnRBOHA* and *NnRBOHB* were significantly upregulated, whereas the remaining *NnRBOH* family genes showed no significant changes in expression. These results indicate that different members of the *NnRBOH* family may play distinct roles during the heat-stress response. Collectively, this study clarifies the evolutionary characteristics of the *NnRBOH* family and identifies *NnRBOHA* as a potential key candidate gene for heat-tolerance genetic improvement, providing clear targets for subsequent functional validation and molecular breeding.

## 1. Introduction

Reactive oxygen species (ROS) are crucial signaling molecules in plant cells, playing key roles in plant growth and development, responses to biotic and abiotic stresses, and programmed cell death [1,2]. Respiratory burst oxidase homologs (RBOHs), also known as NADPH oxidases, are the key enzymes responsible for ROS production [3,4]. Originally discovered in phagocytes of the human immune system, these enzymes are primarily found in mammalian neutrophils and plant cells [5]. Plant RBOHs are homologous to the mammalian gp91phox [6] and typically contain six conserved transmembrane domains, two heme groups, a C-terminal FAD and NADPH-binding domain, and an N-terminal specific EF-hand calcium-binding domain [7,8]. This domain architecture enables plant RBOHs to transfer electrons from cytosolic NADPH to extracellular molecular oxygen, generating superoxide anion (O^2−^), which is then rapidly dismutated to hydrogen peroxide (H_2_O_2_) by superoxide dismutase [9].

Accumulating evidence suggests that RBOH-mediated ROS production plays multiple roles in plant growth and development, hormone signaling, and stress responses [10,11,12]. In terms of growth and development, RBOHs regulate processes such as root hair tip elongation, pollen tube growth, and stomatal movement [13,14]. For example, *AtRBOHC* in *Arabidopsis thaliana* is involved in polar root hair growth, while *AtRBOHH* and *AtRBOHJ* play specific roles in pollen tube tip growth [15]. Regarding hormone signaling, RBOHs are tightly associated with abscisic acid (ABA), jasmonic acid (JA), salicylic acid (SA), and other phytohormone pathways [16,17]. In particular, they mediate ABA-induced stomatal closure under drought stress [18]. Regarding stress responses, RBOHs serve as a central hub in plant stress signal transduction networks [10]. Upon pathogen infection, the RBOH-mediated oxidative burst directly inhibits pathogen invasion and activates downstream defense genes, promoting callose deposition to reinforce cell walls [19]. Furthermore, various abiotic stresses, including drought, salinity, and extreme temperatures, trigger RBOH expression and ROS accumulation to initiate adaptive responses [20,21,22].

Given the functional importance of RBOHs, this gene family has been identified and characterized at the genome-wide level across a diverse range of plant species. In *A*. *thaliana*, ten *AtRBOHs* have been identified and classified into constitutively expressed and tissue-specific types based on their expression patterns and functional roles [23]. In *Oryza sativa*, nine *OsRBOHs* exhibit differential expression across various tissues and in response to abiotic stresses, with high temperature up-regulating *OsNox5–9* but significantly down-regulating *OsNox1–3* and *OsFRO1* [24]. The *Citrus sinensis* genome contains seven *CsRBOHs*, among which *CsRBOHD* plays a critical role in cold stress responses [25]. In *Nicotiana tabacum*, fourteen *NtRBOH* members have been identified, among which *NtRBOHD* and *NtRBOHE* were significantly down-regulated under cold and drought stress, suggesting a negative response of these genes to certain abiotic stresses [26]. In *Gossypium hirsutum*, twenty-six *GhRBOHs* show spatiotemporal-specific expression patterns during development and under stress conditions, and among them, a total of nine genes (*GhRBOH3/4/6/9/10/21/22/25/26*) showed continuous and stable expression under high-temperature stress treatment [1]. In *Triticum aestivum*, thirty-nine *TaRBOHs* have been identified, with several members involved in drought and heat stress responses [27]. These findings suggest that RBOHs play conserved but species-specific roles in thermotolerance, yet their functional characterization in many economically important plants remains limited.

*Nelumbo nucifera* is a perennial aquatic herb in the family Nelumbonaceae. It possesses high ornamental, edible, and medicinal values. Its rhizomes, seeds, and leaves are not only traditional medicinal and edible resources but also important components of the aquatic crop economic chain, bringing significant economic benefits to the aquatic crop industry [28]. However, as a typical thermophilic aquatic plant, *N*. *nucifera* frequently suffers from persistent heat stress during the peak growing season in summer, which directly inhibits photosynthesis and dry matter accumulation, leading to inhibited rhizome swelling, reduced yield, and severe deterioration of seed quality and marketability [29]. Although *N*. *nucifera* exhibits strong adaptability to various abiotic stresses such as water stress and pathogen infection [30], the molecular regulatory mechanisms underlying its response to heat stress remain largely unclear, which greatly limits the progress of improving *N*. *nucifera* heat tolerance through molecular breeding approaches. Respiratory burst oxidase homologues (RBOHs) are core catalytic enzymes that produce reactive oxygen species (ROS) and play a pivotal role in stress signal transduction and physiological adaptation in many plant species. However, no systematic genome-wide analysis of this gene family has been reported in *N*. *nucifera*. On this basis, this study performed a systematic bioinformatic identification of *NnRBOH* family members using the published *N*. *nucifera* genome data, and comprehensively analyzed their physicochemical properties, phylogeny, gene structures, conserved motifs, promoter cis-elements, and syntenic relationships. This study aims to reveal the evolutionary characteristics and functional divergence of the *NnRBOH* gene family, and to provide a theoretical foundation for future investigations into the regulatory mechanisms of *NnRBOH*-mediated ROS signaling in *N*. *nucifera* growth, development, and heat stress responses, thereby offering candidate gene resources for breeding heat-tolerant *N*. *nucifera* varieties.

## 2. Results

### 2.1. Identification and Physicochemical Properties of RBOH Gene Family in Nelumbo nucifera

A total of ten non-redundant members of the *RBOH* gene family were identified in the *N. nucifera* genome through HMMER search and BLASTP analysis combined with conserved domain validation. Based on the homology with *A. thaliana RBOHs*, these members were named *NnRBOHA* to *NnRBOHJ* (Table S1).

Analysis of their physicochemical properties revealed that the base pair numbers of NnRBOHs ranged from 2127 to 3369 bp, and the protein lengths varied from 708 to 1122 amino acids (Table S1). Among them, NnRBOHC had the longest length of 1122 aa, while NnRBOHJ had the shortest length of 708 aa. The molecular weights ranged from 80.93 to 124.52 kDa, which were positively correlated with amino acid length. The theoretical isoelectric points (pI) ranged from 6.27 to 9.39, among which NnRBOHH (pI, 6.27) was an acidic protein, whereas the remaining nine members were basic proteins with pI values ranging from 8.02 to 9.39. The instability index ranged from 35.88 to 49.86, with NnRBOHA (35.88) and NnRBOHD (36.04) predicted as stable proteins (instability index < 40), while the others were unstable. The aliphatic index ranged from 79.12 to 95.67, indicating high thermostability. The GRAVY values of all NnRBOHs were negative (ranging from −0.284 to −0.094), suggesting that they are hydrophilic proteins. In silico subcellular localization prediction using Plant-mPLoc indicated that all ten RBOHs are localized to the cell membrane. To further validate this prediction, we performed an additional analysis using WoLF PSORT, which yielded fully consistent results, both supporting their plasma membrane localization.

### 2.2. Phylogenetic Analysis

**Figure 1:**
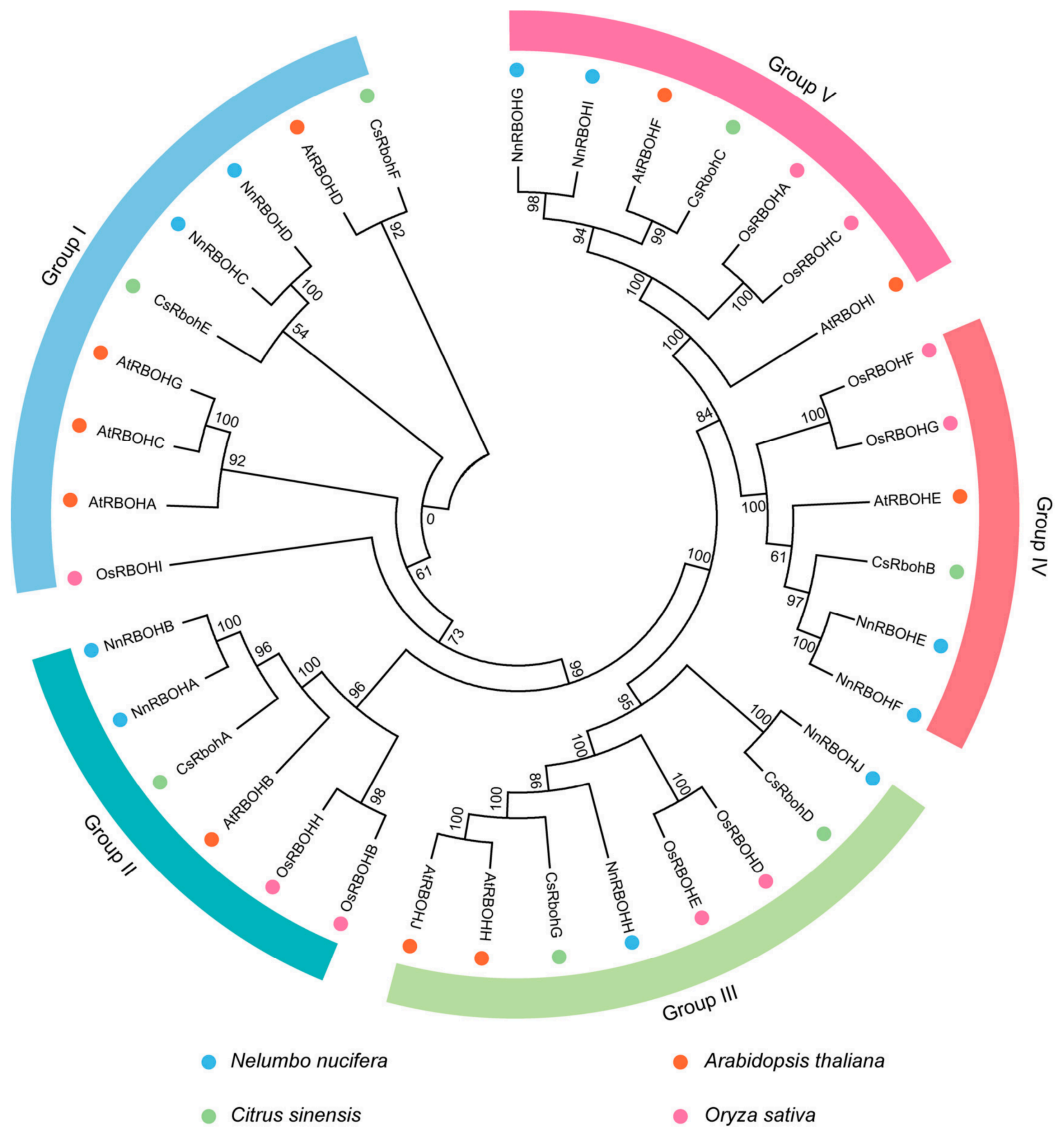
Phylogenetic analysis of RBOHs from *Nelumbo nucifera* (Nn), *Citrus sinensis* (Cs), *Arabidopsis thaliana* (At), and *Oryza sativa* (Os).

To explore the evolutionary relationships among the RBOHs from different species, a phylogenetic tree was constructed using RBOH protein sequences from four species, including *N. nucifera* (10), *A*. *thaliana* (10), *O*. *sativa* (9), and *C*. *sinensis* (7). The results of the phylogenetic analysis showed that all RBOHs were clearly divided into five major subfamilies (Groups I–V) (Figure 1). The ten NnRBOH members, designated NnRBOHA through NnRBOHJ, were distributed across all five subfamilies, suggesting that the NnRBOH gene family has undergone evolutionary diversification, with distinct members potentially acquiring divergent biological functions.

Among the five groups, Group I contained the largest number of RBOH members (9), followed by Group III (8), Group V (7), Group II (6), and Group IV (6). Specifically, Group I consisted of two proteins, NnRBOHC and NnRBOHD; Group II consisted of NnRBOHA and NnRBOHB; Group III consisted of NnRBOHH and NnRBOHJ; Group IV consisted of NnRBOHE and NnRBOHF; and Group V consisted of NnRBOHG and NnRBOHI. Notably, each of the five groups contained at least one member from *N. nucifera*, *A. thaliana*, *O*. *sativa*, and *C*. *sinensis*, indicating that the *RBOH* gene family originated prior to the divergence of monocots and dicots and has maintained a high degree of evolutionary conservation.

### 2.3. Chromosomal Localization Analysis

**Figure 2:**
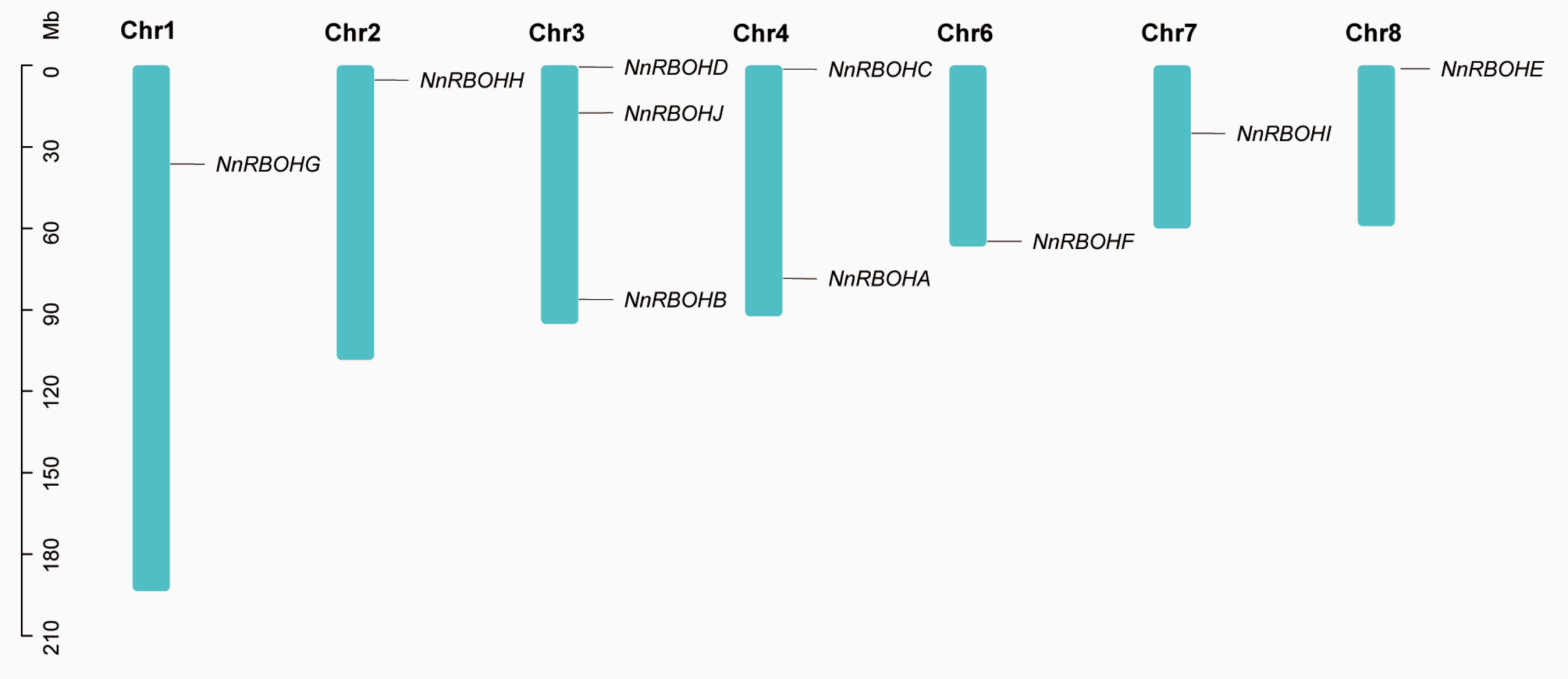
Chromosomal localization map of *NnRBOHs*.

Chromosomal localization analysis revealed that the 10 *NnRBOHs* were unevenly distributed on eight chromosomes of *N. nucifera* (Figure 2). *NnRBOHG* was localized on chromosome 1, *NnRBOHH* on chromosome 2, *NnRBOHD*, *NnRBOHJ*, and *NnRBOHB* on chromosome 3, *NnRBOHC* and *NnRBOHA* on chromosome 4, *NnRBOHF* on chromosome 6, *NnRBOHI* on chromosome 7, and *NnRBOHE* on chromosome 8.

### 2.4. Gene Structure Analysis and Cis-Acting Element Analysis

**Figure 3:**
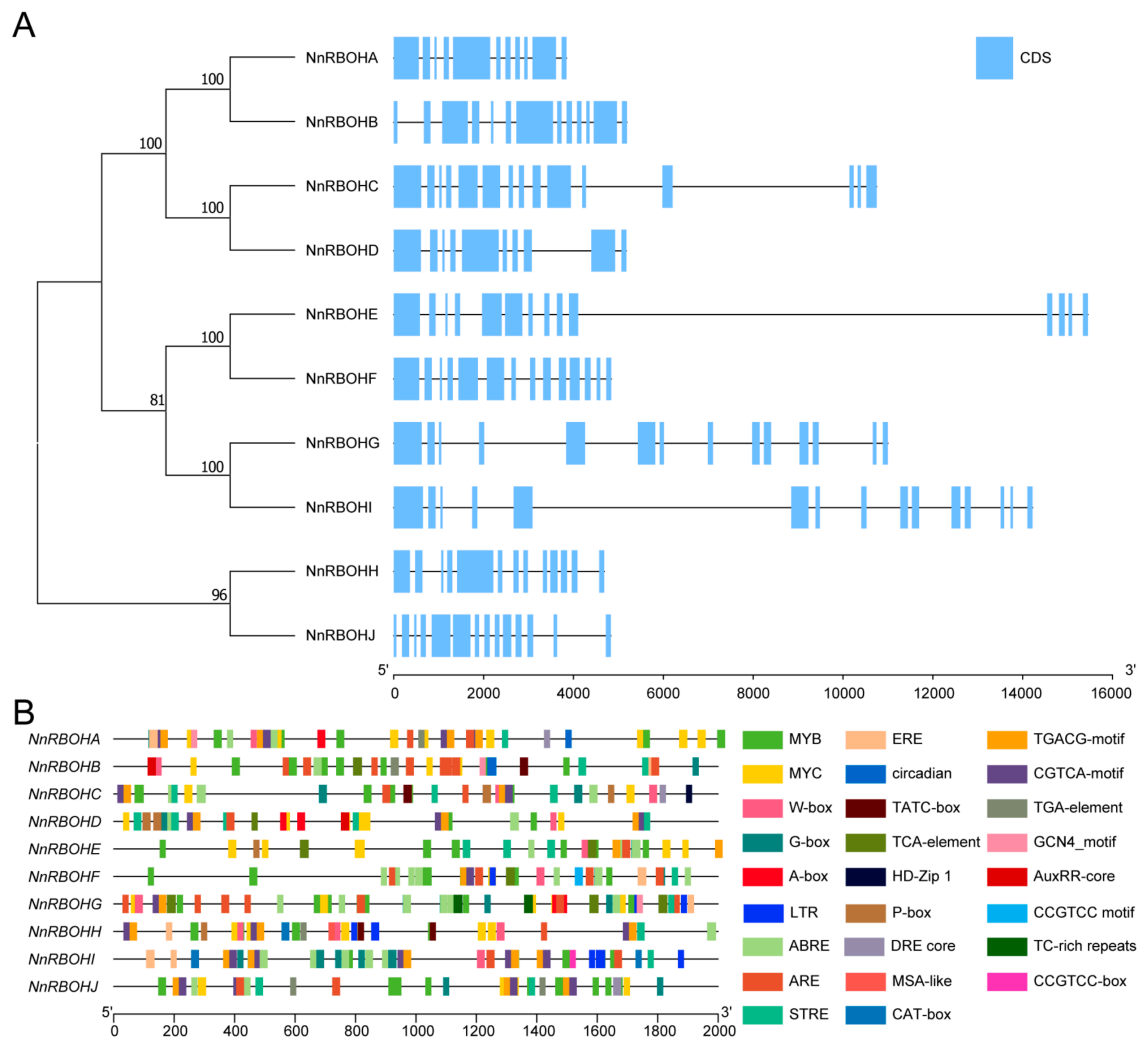
Gene structure and cis-acting element analysis of *NnRBOHs*: (**A**) Schematic diagram of exon-intron structure of *NnRBOHs*. (**B**) Schematic diagram of cis-acting element distribution in the promoter region of *NnRBOHs*.

The gene structure analysis showed that the exon-intron organization patterns of the ten *NnRBOHs* (from *NnRBOHA* to *NnRBOHJ*) exhibited notable differences; however, the structural features of members within the same phylogenetic branch displayed marked similarities, suggesting a close evolutionary relationship among them (Figure 3A). All *NnRBOHs* contained complete coding sequences (CDS), and the number of exons showed obvious variation, ranging from 10 (*NnRBOHD*) to 14 (*NnRBOHE*, *NnRBOHF*, *NnRBOHG*, *NnRBOHH*, *NnRBOHI*, and *NnRBOHJ*). Most *NnRBOHs* contained 14 exons (*NnRBOHE*, *NnRBOHF*, *NnRBOHG*, *NnRBOHH*, *NnRBOHI*, and *NnRBOHJ*), reflecting the structural diversity of the gene family. In terms of intron composition, the members exhibited variability in both the number and types of introns, among which *NnRBOHE*, *NnRBOHF*, *NnRBOHG*, *NnRBOHH*, *NnRBOHI*, and *NnRBOHJ* contained the highest number of introns, while *NnRBOHD* contained the fewest.

Cis-acting elements are known to play vital roles in the regulation of gene expression. To better understand the function and transcriptional regulation mechanism of *NnRBOHs*, a total of twenty-six types of cis-acting elements were identified within the 2 kb region upstream of the translation start site of each *NnRBOH* gene. The results showed (Figure 3B) that the promoter regions of all *NnRBOHs* were rich in various functional elements, which could be classified into three major categories: stress-responsive elements (including MYB, MYC, W-box, STRE, DRE core, TC-rich repeats, LTR, ARE, and GCN4_motif involved in drought, low temperature, and defense responses), hormone-responsive elements (such as ABRE, ERE, TGA-element, AuxRR-core, CGTCA-motif, TGACG-motif, P-box, TCA-element, and TATC-box associated with abscisic acid, ethylene, auxin, methyl jasmonate, gibberellin, and salicylic acid signaling), and development-related elements (including G-box, A-box, CCGTCC motif, CCGTCC-box, CAT-box, HD-Zip 1, MSA-like, and circadian involved in light signaling, tissue differentiation, and circadian regulation). Among these, MYB, MYC, TATC-box, and ABRE were highly enriched in the promoter regions of most *NnRBOHs*.

### 2.5. Analysis of Protein Structural Features

**Figure 4:**
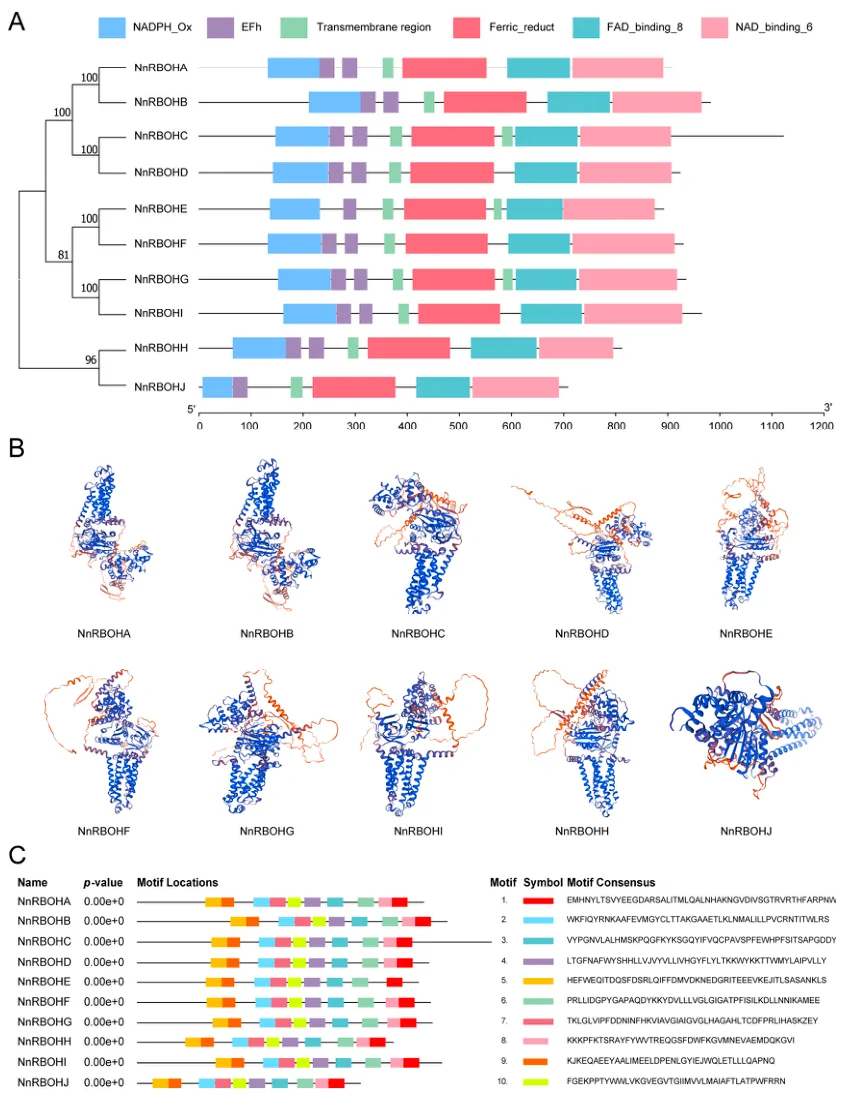
Domain composition and conserved motif analysis of NnRBOHs: (**A**) Domain composition of NnRBOHs. (**B**) Predicted tertiary structures of NnRBOHs. (**C**) Distribution of ten conserved motifs in NnRBOHs.

Multiple sequence alignment revealed the presence of characteristic conserved domains in the NnRBOHs. The results showed that all ten NnRBOHs encoded proteins containing the core domains typical of plant RBOHs, and the arrangement of these domains was highly conserved from the N-terminus to the C-terminus, appearing in the following order: EF-hand calcium-binding domain (EFh), ferric reductase domain (Ferric_reduct), transmembrane region, NADPH oxidase core domain (NADPH_Ox), FAD-binding domain (FAD_binding_8), and NAD-binding domain (NAD_binding_6) (Figure 4A). The presence of the EF-hand calcium-binding domain suggests that the activity of NnRBOHs may be regulated by Ca^2+^, while the conserved distribution of the transmembrane region is consistent with subcellular localization predictions, further supporting their functional localization to the plasma membrane. The integrity of the NADPH_Ox, FAD_binding_8, and NAD_binding_6 domains indicates that NnRBOHs possess typical NADPH oxidase activity, enabling the transfer of electrons from cytosolic NADPH to extracellular oxygen, thereby generating ROS.

To further explore the structural characteristics of NnRBOHs, homology modeling was employed to determine the three-dimensional structures of the 10 NnRBOHs using the Swiss-Model web server. The results showed that all NnRBOHs exhibited highly similar tertiary structures, each consisting of an N-terminal cytoplasmic domain and a C-terminal transmembrane domain (Figure 4B). The N-terminal cytoplasmic domain mainly contained the EF-hand calcium-binding domain and the ferric reductase domain, displaying a typical α-helical folding pattern. The C-terminal transmembrane domain consisted of six conserved transmembrane α-helices, forming a transmembrane channel into which the NADPH oxidase core domain, FAD-binding domain, and NAD-binding domain were embedded.

Through sequence alignment and conserved motif analysis, a total of 10 highly conserved motifs (Motif 1–10) were identified, all with *p*-values of 0.00 × 10^0^, indicating the statistical significance of their conservation (Figure 4C). The presence of all ten conserved motifs in the same sequential order across all ten NnRBOH members strongly indicates the high evolutionary conservation of this gene family. Combined with domain annotation information, it was determined that in the NnRBOHs, Motif 5 is primarily located in the N-terminal NADPH_Ox domain region; Motifs 2, 4, 7, and 10 together constitute the Ferric_reduct domain; Motifs 3 and 6 are located within the FAD_binding_8 domain; and Motifs 1, 6, and 8 form the C-terminal NAD_binding_6 domain.

### 2.6. Synteny Analysis

**Figure 5:**
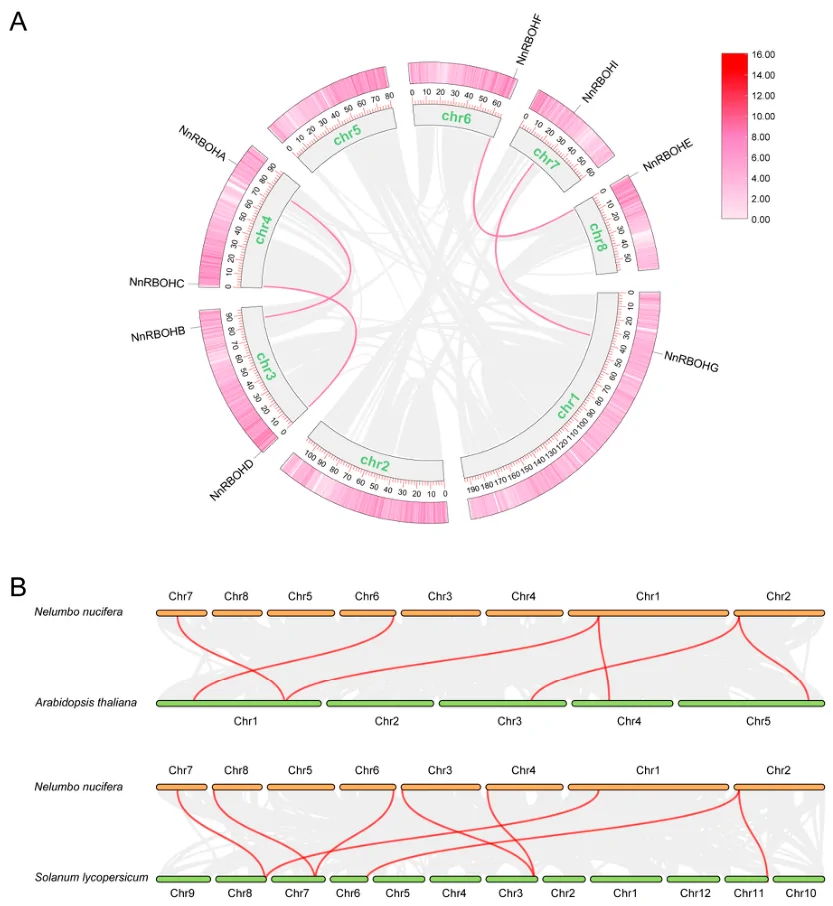
Synteny analysis of *NnRBOHs*: (**A**) Intraspecific synteny analysis of *NnRBOHs*. (**B**) Interspecific synteny analysis of *RBOHs* between *Nelumbo nucifera* and *Arabidopsis thaliana*/*Solanum lycopersicum*.

To elucidate the evolutionary expansion pattern of the *NnRBOH* gene family, we performed an intraspecific synteny analysis. The results (Figure 5A) showed that eight *NnRBOHs* were localized on eight chromosomes (Chr1–Chr8), although their distribution across the chromosomes was uneven. Specifically, they were distributed on Chr1 (*NnRBOHG*), Chr3 (*NnRBOHB* and *NnRBOHD*), Chr4 (*NnRBOHA* and *NnRBOHC*), Chr6 (*NnRBOHF*), Chr7 (*NnRBOHI*), and Chr8 (*NnRBOHE*), whereas no *NnRBOHs* were detected on Chr2 or Chr5. In the circular layout diagram, multiple chromosomes were connected by pink lines, with each line representing a pair of homologous genes. Notably, significant collinear relationships were observed between *NnRBOHA* and *NnRBOHB*, *NnRBOHC* and *NnRBOHD*, *NnRBOHE* and *NnRBOHF*, and *NnRBOHI* and *NnRBOHG*.

To further investigate the evolutionary conservation of the *NnRBOH* gene family, we performed interspecific synteny analysis between *N. nucifera* and *A*. *thaliana* as well as tomato. The results (Figure 5B) showed that a total of six homologous gene pairs were identified between *N. nucifera* and *A. thaliana*, located on *N. nucifera* chromosomes Chr1, Chr2, Chr6, and Chr7. Between *N. nucifera* and tomato, eight homologous gene pairs were detected, distributed across seven chromosomes (Chr1, Chr2, Chr3, Chr4, Chr6, Chr7, and Chr8). In comparison, the syntenic relationship between *N. nucifera* and tomato was closer. Further analysis revealed that the syntenic *RBOH* genes in *N. nucifera* were widely distributed across seven chromosomes, whereas no *RBOH* genes were detected on the remaining chromosome (Chr5). These findings provide important insights into the evolutionary dynamics of the *RBOH* gene family across different species.

In *A. thaliana*, synteny analysis revealed collinear relationships between three *NnRBOH* genes (*NnRBOHF*, *NnRBOHG*, and *NnRBOHI*) and their corresponding genes in *A. thaliana* (Figure 5B). Notably, *NnRBOHG* was found to be associated with two collinear gene pairs in *A. thaliana*. No collinear relationships were detected between *A. thaliana* and *NnRBOHA*, *NnRBOHB*, *NnRBOHC*, *NnRBOHD*, or *NnRBOHE*. In tomato, all eight *NnRBOH* genes exhibited collinear relationships with genes in *S. lycopersicum*.

### 2.7. Expression Analysis of NnRBOH Genes

**Figure 6:**
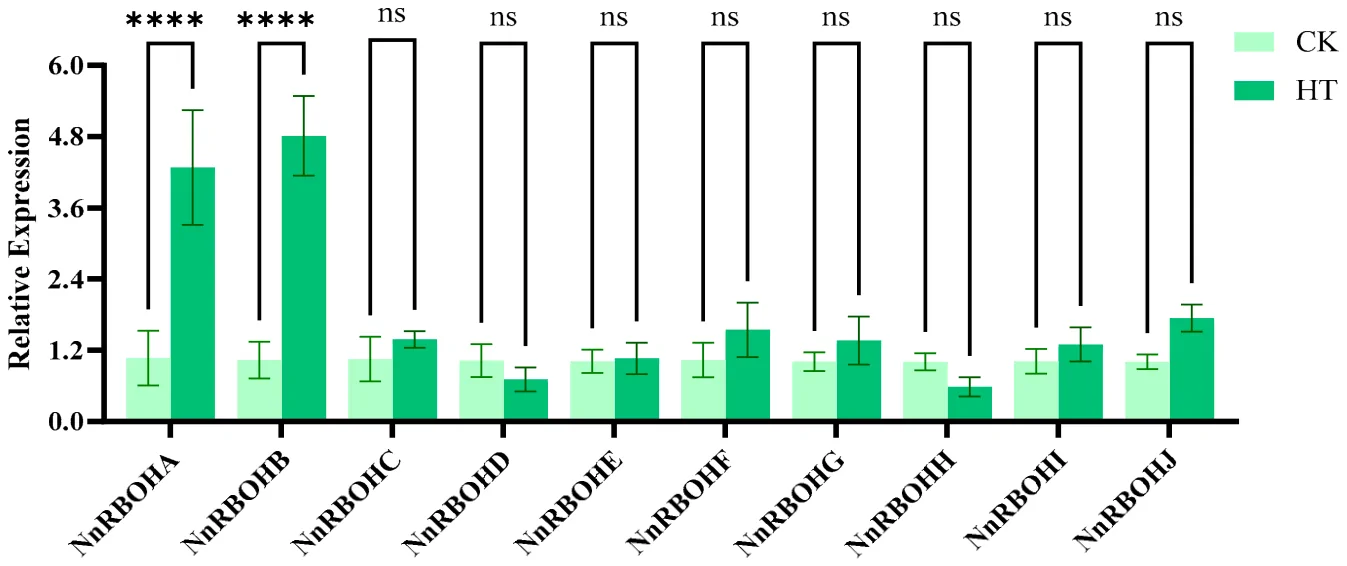
Expression levels of *NnRBOHs* under control and high-temperature conditions. CK, control (normal temperature); HT, high-temperature treatment. Asterisks indicate significant differences among groups (**** *p* < 0.0001; ns, not significant).

qRT-PCR was performed using RNA isolated from *N. nucifera* tissues to analyze the expression patterns of ten *NnRBOH* family genes under normal control (CK) and high temperature stress (HT) conditions (Figure 6). Significant transcriptional responses to high temperature were only detected in *NnRBOHA* and *NnRBOHB*. The two genes showed extremely significant upregulation under high-temperature treatment, with their relative expression levels obviously higher than those in the control group, and the difference reached an extremely significant level. For the remaining eight *NnRBOH* family members including *NnRBOHC*, *NnRBOHD*, *NnRBOHE*, *NnRBOHF*, *NnRBOHG*, *NnRBOHH*, *NnRBOHI* and *NnRBOHJ*, there were no statistically significant differences in gene expression between the control and high-temperature treatment groups. This indicates that *NnRBOHA* and *NnRBOHB* respond to high temperatures and may play an important role in high-temperature stress.

### 2.8. Silencing of NnRBOHA in Nelumbo nucifera by VIGS

**Figure 7:**
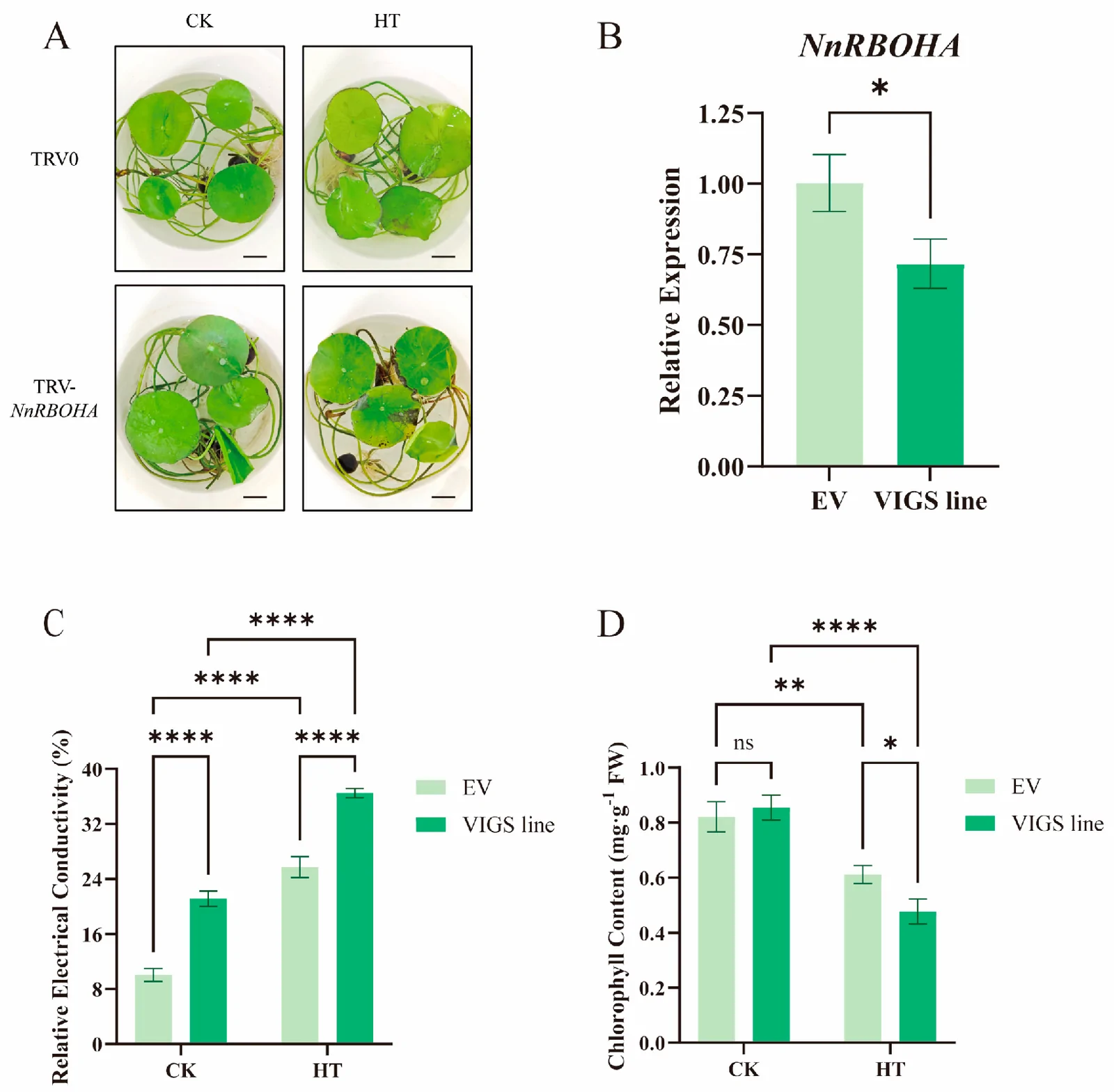
Silencing of *NnRBOHA* by VIGS reduces heat tolerance in *Nelumbo nucifera*: (**A**) Phenotype. Scale bar = 2 cm. (**B**) qRT-PCR validation for the silencing efficiency of *NnRBOHA*. (**C**) Relative electrolyte conductivity. (**D**) Chlorophyll content. Asterisks indicate significant differences among groups (* *p* < 0.05, ** *p* < 0.01, **** *p* < 0.0001; ns, not significant).

Since *NnRBOHA* was significantly induced under heat stress and its promoter region contains multiple stress-responsive elements, we selected this gene for further functional analysis via virus-induced gene silencing (VIGS) in *N. nucifera*. In the VIGS lines (TRV-*NnRBOHA*), the transcript level of *NnRBOHA* was significantly reduced compared with that in the empty vector control (TRV0) (Figure 7B). Under normal growth conditions, no obvious phenotypic differences were observed between the TRV0 control plants and the VIGS lines; however, after high-temperature treatment, the VIGS plants exhibited leaf yellowing and severe brown spotting (Figure 7A), increased relative electrolyte leakage (Figure 7C), decreased chlorophyll content (Figure 7D), and an overall poorer growth phenotype than the TRV controls. These results indicate that the silencing of *NnRBOHA* in *N. nucifera* compromised heat tolerance.

## 3. Discussion

Respiratory burst oxidase homologs (RBOHs) are the main source of ROS production in plants and are therefore involved in regulating diverse biological processes, including growth, development, and stress responses [31]. However, a systematic analysis of the *RBOH* gene family in *N*. *nucifera* has not yet been reported. In this study, a total of ten *RBOH*s were identified from the *N. nucifera* genome (Table S1). This number is similar to those in *A*. *thaliana* (10) [23] and *O*. *sativa* (9) [24], but higher than that in *C*. *sinensis* (7) [25], indicating that the *RBOH* gene family is conserved across different species while also undergoing species-specific expansion. Subcellular localization prediction showed that all NnRBOHs are localized to the plasma membrane (Table S1), which is consistent with findings in other plants [32,33], suggesting that they may play important roles in regulating ROS production. Domain composition, gene structure, and conserved motif analyses indicated that the *NnRBOH* gene family has remained relatively conserved during evolution (Figure 3A and Figure 4).

Phylogenetic analysis divided the RBOHs from *N. nucifera*, *A*. *thaliana*, *O*. *sativa*, and *C*. *sinensis* into five subfamilies (Figure 1), consistent with previously reported classification results [34,35]. The ten *N. nucifera* members were distributed across all five subfamilies, indicating that the *NnRBOH* gene family is evolutionarily diverse and that different members may have diverged to acquire distinct biological functions. Furthermore, all five subfamilies contained members from both monocots (*O*. *sativa*) and dicots (*N. nucifera*, *A*. *thaliana*, *C*. *sinensis*), indicating that the *RBOH* gene family originated before the divergence of monocots and dicots and has maintained high evolutionary conservation. This implies that the core biochemical function of RBOH-dependent ROS production is indispensable for fundamental plant biological processes, including stress sensing and hormone-mediated signal transduction. Meanwhile, the retention of multiple homologs within each subfamily also provides a genetic basis for functional diversification, enabling lineages to adapt to species-specific environmental challenges.

Cis-acting element analysis revealed that the promoter regions of *NnRBOHs* are enriched with various functional elements (Figure 3B), which could be classified into three major categories: stress-responsive, hormone-responsive, and development-related elements [36]. Among these, MYB, MYC, TATC-box, and ABRE were significantly enriched in the promoters of most *NnRBOHs*. ABRE motifs are recognized by ABA-responsive bZIP transcription factors, which mediate abscisic acid-dependent stress signaling pathways. MYB and MYC elements serve as binding sites for MYB/MYC transcription factors, which are central regulators of drought and heat stress responses in plants. In addition, the TATC-box is a typical stress-responsive motif closely associated with plant defense and environmental adaptation. The coexistence of these diverse cis-elements indicates that NnRBOHs may be transcriptionally regulated by multiple upstream transcription factors and participate in complex ABA-dependent and stress-related signaling pathways. This distribution pattern has also been reported in other plants, such as *A*. *thaliana*, *O*. *sativa*, and *G. hirsutum* [1,37], indicating that *RBOHs* are involved in multiple physiological processes, including development, biotic and abiotic stress responses, and hormone signaling. Notably, several elements associated with drought (MYB) and defense responses were identified in the *N. nucifera* promoters, suggesting that *NnRBOHs* may be involved in *N. nucifera* adaptation to multiple stresses.

Expression pattern analysis showed that under high-temperature stress, the ten *NnRBOHs* exhibited distinct differential expression patterns. *NnRBOHA* and *NnRBOHB* were significantly up-regulated, whereas the other eight *NnRBOH* members exhibited no significant changes in transcript levels (Figure 6). In *O*. *sativa*, multiple *RBOHs* are induced by heat, drought, and salt stress; among them, *OsNox5*, *OsNox6*, *OsNox7*, *OsNox8*, and *OsNox9* were significantly up-regulated under high temperature [24]; *OsCML27* integrates phosphorylation and Ca^2+^ signals to regulate drought tolerance of *O. sativa* by fine-tuning *OsRbohB*-mediated ROS signaling [38]. In *G*. *hirsutum*, a total of nine genes (*Ghrboh3*/*4*/*6*/*9*/*10*/*21*/*22*/*25*/*26*) exhibited consistently stable expression under high-temperature stress [1]. Such inter-species comparison suggests partial functional conservation of heat-responsive *RBOH* regulation, yet also highlights lineage-specific divergence. In *N*. *nucifera*, the specific and strong heat-induced up-regulation restricted to *NnRBOHA* and *NnRBOHB* implies that these two paralogs have evolved as the primary *RBOH* candidates for heat-stress signalling, presumably by triggering downstream defence responses through ROS production. The lack of significant transcriptional alteration for other *NnRBOH* members under our heat-treatment conditions does not exclude their biological roles; they might function in other abiotic or biotic stress pathways or act at different developmental stages.

To verify the function of *NnRBOHA* in heat tolerance, we further silenced this gene using VIGS, and found that the VIGS lines exhibited yellowing, severe browning (Figure 7A), higher electrolyte leakage (Figure 7C), lower chlorophyll content (Figure 7D), and overall poorer growth than the TRV control plants under high-temperature treatment, indicating that silencing of *NnRBOHA* compromised heat tolerance in *N*. *nucifera*. Although our results establish a critical role for *NnRBOHA* in the heat stress response, we did not examine whether its silencing affects the transcript levels of other *NnRBOH* family members. Future studies will be needed to investigate potential cross-regulation, compensatory effects, or functional redundancy among these genes, which could provide a more comprehensive understanding of how the *NnRBOH* family collectively coordinates ROS homeostasis and thermal adaptation in *N*. *nucifera*.

## 4. Materials and Methods

### 4.1. Genome Data Sources and Gene Identification

The genomic sequence data, protein sequences, and annotation information of *N. nucifera* were downloaded from the *N. nucifera* genome database (http://106.15.231.155:8001/, accessed on 12 February 2026). To identify *RBOH* gene family members, the sequence alignments of the NADPH oxidase core domain (PF08414), ferric reductase domain (PF01794), FAD-binding domain (PF08022), and NAD-binding domain (PF08030) were obtained from the Pfam database (http://pfam.xfam.org/) and used to build hidden Markov model (HMM) profiles utilizing the hmmbuild program in HMMER 3.2.2 [39]. Subsequently, the hmmsearch program was employed with an E-value cutoff of 1 × 10^−5^ to identify putative NnRBOHs. To avoid missing any members, we additionally performed BLASTP searches against the *A*. *thaliana* RBOH protein sequences using an E-value threshold of 1 × 10^−5^, and redundant protein sequences were discarded. All putative sequences were further confirmed through the Pfam database (http://pfam.xfam.org/) and SMART database (http://smart.embl-heidelberg.de/, accessed on 27 March 2026) for the presence of conserved RBOH domains, ultimately determining the members of the *NnRBOH* gene family [33,40].

### 4.2. Physicochemical Properties and Subcellular Localization Analysis

The physicochemical properties of NnRBOHs, including amino acid length (aa), molecular weight (Da), theoretical isoelectric point (pI), instability index (II), aliphatic index (Ai), and grand average of hydropathicity (GRAVY), were calculated using the ExPASy-ProtParam tool (http://web.expasy.org/protparam/, accessed on 9 April 2026) [41]. The subcellular localization of the NnRBOHs was predicted using the Plant-mPLoc website (http://www.csbio.sjtu.edu.cn/bioinf/plant-multi/, accessed on 21 April 2026) [42] and further verified using the WoLF PSORT website (https://wolfpsort.hgc.jp/, accessed on 27 April 2026) to ensure robustness and cross-validation of the prediction results.

### 4.3. Multiple Sequence Alignment and Phylogenetic Analysis

RBOH protein sequences of *A*. *thaliana*, *O*. *sativa*, and *C*. *sinensis* were downloaded from NCBI (UniProtKB) and aligned with the predicted NnRBOH protein sequences in the MEGA-X program (https://www.megasoftware.net/). The sequence alignment was presented with ESPrit 2.2-ENDscript 1.0 [43]. A phylogenetic tree was constructed based on the alignment in the MEGA-X program using the neighbor-joining method with the p-distance model and 1000 bootstrap replicates [44].

### 4.4. Gene Structure and Cis-Acting Element Analysis

The genomic DNA and complete coding sequence (CDS) of each *NnRBOH* were extracted from the *N. nucifera* genome annotation file, and the intron-exon structure of each *RBOH* was predicted using the Gene Structure Display Server (GSDS) v2.0 (http://gsds.cbi.pku.edu.cn) [45]. The 2000 bp region upstream of the translation start site of each *NnRBOH* was extracted from the *N. nucifera* genome as the promoter region and submitted to the PlantCARE database for prediction of cis-acting elements, which were categorized into stress response, hormone regulation, and development-related elements.

### 4.5. Conserved Motif and Domain Analysis

Conserved motifs in the NnRBOHs were predicted using the MEME Suite (http://meme-suite.org/). The analysis parameters were configured to identify the top 10 conserved motifs, whereas the remaining settings were kept at their default [46]. The domain locations were annotated using the SMART database and Pfam database. To determine the three-dimensional structures of the RBOHs, homology modeling was employed using the Swiss-Model web server (https://swissmodel.expasy.org/). Subsequently, the quality of the modeled structures was assessed considering the Ramachandran Plot, stereochemistry (MolProbity score), and clash score parameters (https://swissmodel.expasy.org/assess, accessed on 26 May 2026).

### 4.6. Synteny and Gene Duplication Analysis

Using the Multiple Collinearity Scan toolbox (MCScanX) program [47], synteny blocks within the *N. nucifera* genome were analyzed based on whole-genome protein sequence alignments to identify collinear pairs of *Nn*RBOH*s*. To further reveal the evolutionary conservation of this family, cross-species synteny analysis was performed between *N. nucifera* and the model plants *A*. *thaliana* and tomato, and the results were visualized using the Dual Synteny Plotter in TBtools V2.136 [48]. Finally, the non-synonymous substitution rate (Ka), synonymous substitution rate (Ks), and their ratio (Ka/Ks) for each homologous gene pair were calculated using TBtools to evaluate the selection pressure during evolution [49].

### 4.7. Plant Material and Treatment

The experimental material is the *N. nucifera* cultivar ‘Jinfurong No. 3′, a crucial resource of *N. nucifera*, cultivated under natural conditions at the Aquatic Vegetable Experimental Base of Yangzhou University (Yangzhou, China). The plants were grown under two different temperature treatments: 28/20 °C (Control) or 38/28 °C (Heat), with light/dark cycles. We performed a series of initial experiments at different temperatures to analyse plant growth status. Samples of the leaves were taken 6 h after applying the stresses.

### 4.8. Expression Analysis of NnRBOHs

RNA-seq data and qRT-PCR methods were used to determine the expression profiles of *Nn*RBOH*s* and their response to high-temperature stress conditions. RNA-seq data were downloaded from the NCBI website. The bar chart of gene expression was constructed by prism10.1.2 software.

Total RNA was extracted from leaves subjected to different temperature treatments using the RNA Easy Fast Plant Tissue Kit (Tiangen, Beijing, China). The RNA concentration was determined using a spectrophotometer. First-strand cDNA synthesis was carried out using the TransScript II One-Step gDNA Removal and cDNA Synthesis Super Mix Kit (Vazyme, Nanjing, China). qRT-PCR was performed using a Light Cycle 96 real-time PCR system with the Hieff^®^ qPCR SYBR Green Master Mix Kit (Yeasen, Shanghai, China). The PCR program was as follows: 95 °C for 3 min for pre-denaturation, followed by 15 s at 95 °C for denaturation, 30 s at 60 °C for annealing, and 20 s at 72 °C for extension, with 40 cycles. A melting curve analysis was conducted from 60 to 95 °C. *NnActin* was used as the internal reference gene, and relative gene expression levels were calculated using the 2^−ΔΔCt^ method. All experiments were conducted with three biological replicates. Primers were designed by Primer Premier 6 software (Table S2) and their specificity was verified by NCBI. The same qRT-PCR method described above was also applied to determine the silencing efficiency of *NnRBOHA* in VIGS-treated *N. nucifera* plants. The raw qRT-PCR data for the expression of *NnRBOHs* under CK and HT conditions in *Nelumbo nucifera* are provided in Table S3.

### 4.9. Generation of VIGS Plants

VIGS-mediated suppression of *NnRBOHA* was conducted according to previous protocols [50,51]. A 300 bp fragment of *NnRBOHA* was amplified and inserted into the *SmaI* and *BamHI* sites of tobacco rattle virus-based vector 2 (TRV2) to obtain the pTRV2-*NnRBOHA* construct. The vectors pTRV1, pTRV2, and pTRV2-*NnRBOHA* were introduced into *Agrobacterium tumefaciens* strain GV3101 by heat shock. Briefly, plasmid DNA was added to the competent cells, mixed gently, and incubated on ice for 10 min, followed by liquid nitrogen for 5 min, a 37 °C water bath for 5 min, and then placed on ice for another 5 min. After heat shock, 900 µL of LB medium was added, and the cells were recovered at 28 °C for 2–3 h with shaking, followed by plating on LB agar plates containing the appropriate antibiotics (kanamycin and rifampicin) to select positive transformants. For agroinfiltration, *Agrobacterium* cells carrying pTRV1 were grown to an OD_600_ of 1.0 and then mixed with cells carrying pTRV2-*NnRBOHA* or empty pTRV2 at a 1:1 volume ratio in MES infiltration buffer (10 mM MgCl_2_, 200 mM acetosyringone, and 10 mM MES, pH 5.6) [52]. The mixture was kept in the dark for 4 h at room temperature prior to infiltration into the fully expanded young leaves of *N*. *nucifera* seedlings. Each treatment was repeated three times independently as biological replicates. After infiltration, the seedlings were maintained hydroponically and placed in a light incubator at 20–28 °C and 28–38 °C, respectively, for further observation.

### 4.10. Measurement of Relative Electrolyte Leakage and Chlorophyll Content

Relative electrolyte leakage was determined by measuring the conductivity of leaf discs in distilled water before and after boiling, and expressed as a percentage of total conductivity [53]. Chlorophyll content was extracted with 80% acetone and quantified spectrophotometrically at 663 and 645 nm, with total chlorophyll calculated according to Arnon [54]. Three biological replicates were performed for each treatment.

### 4.11. Statistical Analysis

Three biological and technical replicates were used for each control and treatment sample. Two-way analysis of variance (ANOVA) followed by appropriate multiple comparison tests was performed to validate the expression differences in the RBOHs in the different treated conditions. The *p*-value cutoff ≤ 0.05 was considered to be a statistically significant test result.

## 5. Conclusions

In this study, the ten *NnRBOHs* were identified in the *N*. *nucifera* genome, unevenly distributed across eight chromosomes. Phylogenetic analysis classified these genes into five subfamilies, with members of the same subfamily sharing similar gene structures and conserved motifs. Promoter cis-acting element analysis revealed that the promoter regions of *NnRBOHs* contain multiple elements associated with stress responses, hormone signaling, and developmental regulation. Synteny analysis identified multiple collinear gene pairs within the *NnRBOH* gene family, and revealed a closer collinear relationship with tomato than with *A*. *thaliana*, providing insights into the evolutionary expansion of this family. Expression analysis demonstrated that *NnRBOHA* and *NnRBOHB* were significantly upregulated under high-temperature stress, suggesting their potential key roles in the response of *N*. *nucifera* to high-temperature stress. VIGS silencing experiments confirmed that knockdown of *NnRBOHA* led to reduced transcript levels, increased relative electrolyte leakage, and decreased chlorophyll content, collectively indicating compromised physiological performance under heat stress. These results preliminarily verify the positive regulatory role of *NnRBOHA* in heat tolerance. Accordingly, we propose that *NnRBOHA* mediates ROS-dependent signaling cascades that activate downstream heat shock responses, thereby contributing to thermotolerance in *N*. *nucifera*. Collectively, this study systematically characterizes the molecular evolutionary features and expression patterns under high-temperature stress of the *NnRBOH* gene family, providing a solid foundation for future functional studies and for the application of these candidate genes in heat tolerance breeding of *N*. *nucifera*. Given that stable transformation and CRISPR/Cas9 knockout systems are not yet well-established in *N*. *nucifera*, further functional validation could be prioritized via heterologous expression in model plants and more comprehensive VIGS-based phenotypic and biochemical assays to consolidate the heat-tolerance functions preliminarily observed from VIGS experiments.

## Data Availability

The original contributions presented in this study are included in the article/Supplementary Material. Further inquiries can be directed to the corresponding author.

## Supplementary Materials

The following supporting information can be downloaded at https://www.mdpi.com/article/10.3390/ijms27188366/s1.

## Abbreviations

The following abbreviations are used in this manuscript:

RBOH	Respiratory Burst Oxidase Homolog
ROS	Reactive Oxygen Species
NADPH	Nicotinamide Adenine Dinucleotide Phosphate
CK	normal control (referring to the non-treated control under stress experiments)
HT	high temperature (the heat stress treatment used in our expression analysis)
